# Thrivaad: A Multilingual, Predictive Eye-Sign-Based AAC System Powered by Optimized Deep Learning

**DOI:** 10.3390/s26144503

**Published:** 2026-07-15

**Authors:** Rajesh Kannan Megalingam, Sakthiprasad Kuttankulangara Manoharan, Dhilna Cheriyan Manjooran, Dhanaraj Kamble

**Affiliations:** Humanitarian Technology (HuT) Labs, Department of Electronics and Communication Engineering, Amrita Vishwa Vidyapeetham, Amritapuri, Kollam 690525, Kerrla, India; sakthiprasadkm@am.amrita.edu (S.K.M.); dhilnacm@am.amrita.edu (D.C.M.); dhanarajkamble@am.amrita.edu (D.K.)

**Keywords:** Brain–Computer Interaction (BCI), graphical user interface (GUI), Thrivaad, Netravad, Augmentative and Alternative Communication (AAC)

## Abstract

Around 1.5% of the global population is suffering from speech impairments; the major causes for this are cerebral palsy and ALS, and the only way for these individuals to communicate is through Augmentative and Alternative Communication (AAC). These systems are either electronic or non-electronic. Based on new study developments, electronic methods, such as Brain–Computer Interaction (BCI) and eye-gaze-based communication, are assessed as the best choices, but they have their own limitations, incorporating limited adaptability to changing conditions, such as setup variations and user fatigue, which reduces the system‘s robustness. Our previous study, Netravad, shows potential for addressing these gaps, but it lacks multilingual support and will not yield the same results under changing lighting conditions. This study, Thrivaad, provides multilingual support and text prediction and integrates optimized deep learning to accurately capture eye movements even in varying environmental lighting conditions. Thrivaad uses eye movements as input from a webcam, and the Optuna-optimized YOLOv5 model is used to detect the eye direction accurately. Then communication is established in English, Malayalam, and Hindi. The text-prediction feature of this system improves communication by reducing the number of eye gestures required to form a message. This study included a total of 60 participants across three age groups with 35,263 eye-sign images collected. With this data, the YOLOv5 model is trained and then optimized by Optuna. The proposal system provides accurate eye direction, text prediction, multilingual support, and improved adaptability to changing conditions for eye-based AAC.

## 1. Introduction

Over 3 billion people are living their lives with neurological disorders as of 2021, and these neurological conditions are the main contributors to ill health and disability worldwide. The disability rate has been scaled to 43.1% of the global population, according to the Global Burden of Disease Study. This is 18% higher compared to 1990 [1]. Out of the top ten contributors to nervous system health loss, stroke, dementia, Alzheimer’s, epilepsy, autism, and neonatal encephalopathy will severely impair speech and communication [1]. All India Institute of Speech and Hearing (AIISH) researchers conducted a door-to-door prevalence study in the Andaman Islands. A total of 30,307 residents of towns in north and central Andaman were surveyed, and it was found that among those referred for screening, 47.76% & 52.24% had ear- and speech-related disorders, respectively, indicating the need for communication support in these regions [2]. To communicate with people with severe speech and motor impairments, an advanced system, Augmentative and Alternative Communication (AAC), is a better option [3], but it has its own limitations, such as slow communication speed, limited vocabulary, limited adaptability, and limited contextual word prediction. The study also found that a very small number of word predications compared to natural speech words were counted at 150–250 words per minute. Thus, improving the AAC system is of the utmost priority to enhance the quality of life for people with speech and motor impairments by adding developments such as automating system setup for ease of adaptation, adding context-aware capabilities, and reducing communication latency [3,4,5].

There are multiple systems in research, from basic-level systems like picture boards to fully advanced systems like speech-generating devices (SGDs) that transform typed words into clear speech, making the conversation livelier with personalized vocabulary customization [3]. Individuals with speech impairments rely on nonverbal communication. For them, hand-gesture-based communication using smart gloves has been developed, but it is limited for motor-impaired patients [6]. The same research lab has developed an assistive device for stroke patients with limited hand movement. This device uses an abduction mechanism, and it is again limited to a particular body part (hand) and needs constant user effort [7]. For motor-impaired patients, such as ALS or cerebral palsy patients, eye-tracking technologies are well-suited, as they use eye-gaze input to build conversations by integrating AAC software into the system [8,9]. In cases of severe motor impairments, head-tracking devices and switch-accessible systems are preferred, as they use minimal voluntary movements to build communication [10]. Though the systems have improved over time, they still demand users’ cognitive and physical effort, and communication efficiency gets reduced by the systems that offer limited contextual accuracy [11]. In eye-tracking technologies, the open technical challenge is the detection of eye movements with subtle and atypical patterns [8]. On top of that, these AAC systems are built for English speakers, which prevents native language speakers from utilizing these technologies [12].

It is good practice to have a well-designed graphical user interface (GUI) for assistive medical devices; otherwise, users cannot navigate and operate them independently [13,14,15,16,17]. To make a perfectly inclusive GUI, key design features such as layout customization, font size adjustment, screen brightness adaptation, and intuitive symbol arrangement are essential so that even non-technical users can operate it efficiently [13,18]. Native-language communication makes the user experience even better, but implementing multilingual support in the system requires attention to detail in areas such as text rendering, script handling, and language switching. So, this leads to design complexity [19,20]. More design features, more complexity: this native-language communication through eye movements has been achieved by the Netravaad system, but it lacks word prediction; hence, users need to put more effort into constructing a message, which makes communication even slower compared to average communication speed [21,22,23]. A word data set library with a user-configurable vocabulary is to be integrated into the system so users can easily communicate using this word-prediction feature, and assistive communication can be enhanced for the diverse population [24].

As mentioned earlier, the Netravaad system has achieved a milestone for communication through eye movements, but it has usability limitations. It requires a lot of effort from the user to construct the message letter by letter using every eye gesture, and it only operates in English. To manage these constraints, this work proposes a new inclusive GUI that helps users communicate faster by predicting text with fewer eye gestures, and the language support has been extended to Indian native languages, Malayalam and Hindi. So, this speeds up communication and reduces user effort. To maintain accuracy, the system uses the YOLOv5 module with hyperparameters tuned by Optuna. This makes it easy to detect atypical and subtle eye movements, thereby making the system more reliable and robust. Native languages Malayalam and Hindi make the system more accessible to users, and a wide variety of communities can utilize this technology in their preferred language.

List of contributions from this work:

- Eye Movement Detection Using YOLOv5 + Optuna Hyperparameter Optimization: A deep-learning model based on YOLOv5 is implemented to detect abnormal eye movements in users with severe motor disabilities and in different age groups with varying lighting conditions to achieve better accuracy in comparison to Netravaad.
- Smart Word Selector for Enhanced Eye-Based AAC: A predictive text system called Smart Word Selector is included in the system to reduce the number of eye gestures required to form a sentence, thus increasing user comfort and reducing physical strain in users with severe motor disabilities like ALS and cerebral palsy.
- Multilingual User-Centric GUI for Eye-Based AAC: A new graphical user interface design facilitates communication in English, Hindi, and Malayalam with high contrast graphics and user-centric gaze optimization, thus increasing accessibility to users with severe motor disabilities and those who speak different languages.

The paper is structured into five sections: Section 2—background of the study; Section 3—design and implementation; Section 4—experiment and results; and Section 5—conclusions and future work.

## 2. Related Works

More recently, considerable development of eye-gaze-based interfaces has enhanced communication assistance for those with serious motor impairments and for speechless people. A web GUI image recognition system introduced in [25] revealed improved convolutional neural networks for the computer understanding of graphic user interfaces and pointed out the importance of the intelligent accessibility of interface for HCI. Several eye-tracking methods have become popular to provide one of the best modes for patients with ALS to communicate and several reviews were put forward to present the advantages, the disadvantages, the applications and the issues related to eye-tracking methods [19,26]. Eye gesture recognition control through the application of a spintronic sensor-based eye gesture control system developed in [27], using a magnetic tunnel junction (MTJ) integrated in a contact lens. This allowed wireless recognition of eye gestures. The developed system seemed impressive; however, practical issues of wearability, susceptibility to interference and size are also reasons to hinder it from being widely used. Another example for further development is the framework for both sensing and control of smart assistive systems by which intelligent control of robotic actions can be achieved based on enhanced sensing capabilities and control algorithms (see [28]). As such, the system in [17] provided a new eye-tracking system integrated with four functions, namely: assistive communication, controlling of the wheelchair, managing the smart home and playing entertainment games.

Various researchers have concentrated on enhancing the effectuality of human interaction with an inexpensive and real-time eye-based interaction system. The system blinkify [29] used an IoT-enabled communication appliance where a set of blinks is converted into a specific message, which provides a low-cost interaction method for users having verbal issues. The limitation of this system is the requirement that the user needs to follow the same blinks, and thus increase effort and exhaustion while interacting. The real-time eye movement-based communication interface presented in [30] increases accuracy by tackling the issue of the head’s movement and avoiding interference from user movement. Moreover, a human adaptive gaze-tracking model presented in [31] examines the fatigue and human performance of the users during the continuous gaze interface use. A smart home interaction framework presented in [32] shows the interaction of those using gaze with the integration of Internet of Things devices for smart home controls. A user intent prediction system based on gaze action with machine-learning approaches to minimize user efforts for efficient communication [33] is also proposed, in which the interaction method is where user intention can be determined from eye movement patterns. Eye-to-text communication in [34] directly converts eye movement to text messages, and a computer vision-based communication platform for people suffering from ALS in [35] showed that a computer vision technique can achieve this interaction without specialized eye-tracking hardware, thereby making interaction very cheap for communication.

More recently, researchers have focused on modern trends in eye-gesture recognition and cross-linguistic communication as well as deep-learning-based methods of human–computer interaction (HCI). Meaningful eye and facial motion features extracted through observation of user movement were proposed in [36], which supports using ocular data in broader senses. As shown in [37], a novel gaze motion gesture recognition system was created using an array of infrared sensors within wearable optical glasses. Support of cross-linguistic communication was also examined, and in [18], it was confirmed that enabling the cross-linguistic function would facilitate and assist communication of disabled people as well as increase social participation. Blink-To-Live system [38] provided a standardized eye-based communicative system using a restricted inventory of eye-based gestures to generate text. An eyeball-tracking communication system demonstrated in [39] shows how eye gestures could be utilized for communication through computer vision. Later, Ref. [20] presented a deep-learning-based eye-writing recognition system which performed even better due to innovative preprocessing and data augmentation methods in recognizing eye movements which support expressive writing through human eyes.

From the existing literature, it can be noted that although improvements in terms of accuracy and usability have been made through previous attempts, the majority still lack flexibility in hardware and multilingual capabilities, rely on calibration, and suffer from poor performance in varying environments. Thus, a necessity arises for an effective multilingual eye-tracking AAC platform.

## 3. Design and Implementation

**Figure 1:**
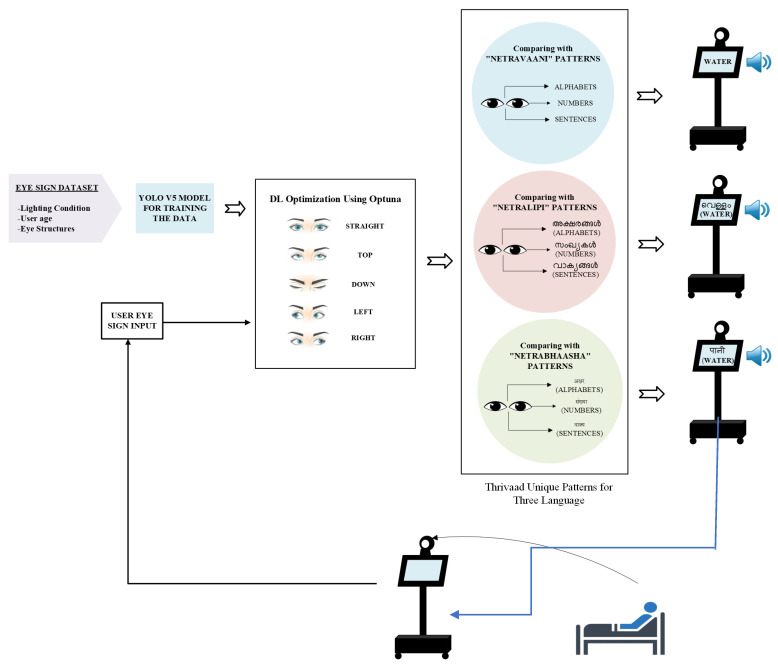
System architecture of Thrivaad.

Thrivaad is an eye-gaze movement-based AAC system. It takes continuous eye movement input and converts it into text and speech output in the preferred language of the user. The live webcam video of eye movement is fed to a deep-learning module, which detects the eye sign (center, left, right, up, and down then it maps them to specified language patterns as per user needs and converts them into sentences, as shown in Figure 1). This system architecture is designed in such a way that it enables smooth nonverbal communication between the system and disabled or motor-impaired patients.

The Thrivaad system architecture, shown in Figure 1, mainly consists of four steps: (1) The eye movement input data collection, where the eye-signs of the user are continuously captured by a web camera mounted on the device. Then this data goes to the next step, (2) the YOLO v5 module, where the eye-sign data collected from the webcam is classified into five different classes (center, left, right, up, and down), but this classification has the knowledge of various lighting conditions and eye patterns of the user with respect to their age differences. So this raw classification is transferred to the next step (3), the deep-learning, Optuna-optimized section: here, the raw classification is refined to an accurate eye-sign classification, since this module is already trained on 35,263 images of various eye signs, so it finally gives the correct eye sign by removing all other noise. (4) Language translation and output: Post-processing the eye-sign data based on the mode selected in the system (auto correct/manual), language (Malayalam, Hindi, English), and output mode (Visuals/voice); the preferred output will be visible on the screen for the user to interact with their preferred options. Since the GUI of this system is advanced, it has three separate dataset modules for native users: NETRAVAANI (English), NETRALIPI (Malayalam), and NETRABHASHA (Hindi). It has letters, numbers, and sentences. The GUI renders these outputs based on the user’s preferred module, and this system has voice output that automates languages accordingly. This process pipeline makes communication effortless and robust.

**Figure 2:**
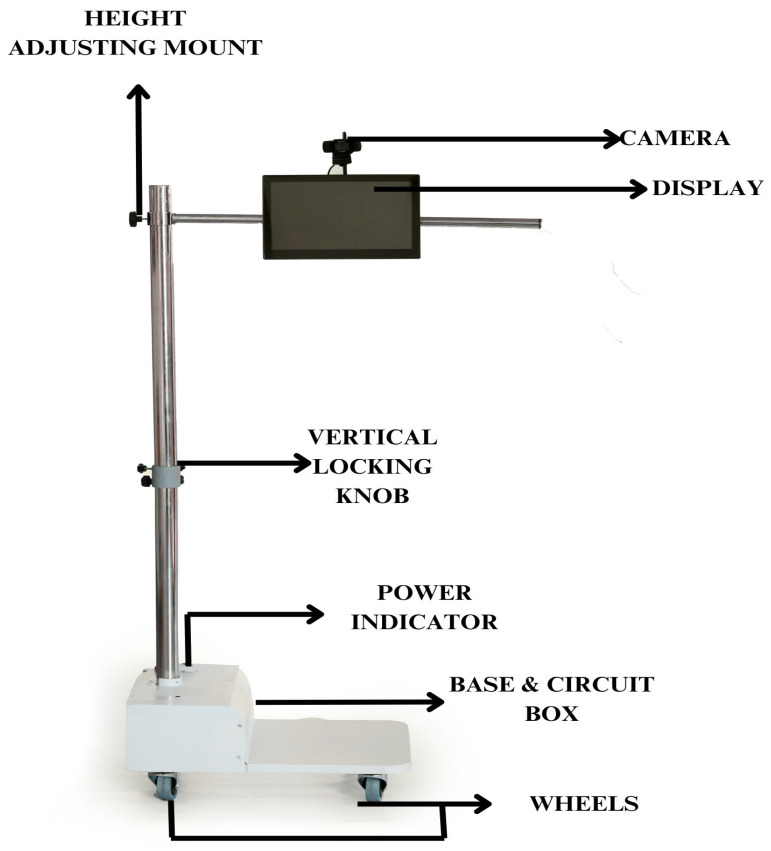
The Thrivaad Device.

The Thrivaad Device, as shown in Figure 2, has a total of 10 components: Web camera, gooseneck flexible metal ring tube, touch display, speaker, connecting wires, hollow vertical tube, sideways bars, locking knobs, base circuit box, and caster wheels. These are connected in the following fashion: The camera is connected to a gooseneck flexible mount and fixed to a stainless-steel bar running sideways, and the touch display is integrated with a speaker and mounted at the same location where the gooseneck is fixed to the side bar, so this entire module can be moved sideways when the patient is in bed. This sidebar is connected to a hollow stainless-steel vertical tube via locking knobs, so the module can be moved up and down when the patient is in a wheelchair. The connecting wires are drawn through the tube and connect to a circuit box in the base box to avoid issues during operation. So, this whole structure stands on four caster wheels for portability.

To make the system robust and adaptable, the dataset was designed to include participants from different age groups and diverse lighting conditions. A total of 60 participants were recruited solely for eye-sign data collection and model validation, distributed across three age groups: 20–40, 40–60, and 60–80 years, with 20 participants in each group. Of the 60 participants, 21 were female and 39 were male. A total of 35,263 eye-sign images were collected from these participants. To evaluate the generalization capability of the proposed model, the dataset was partitioned at the participant level using an 80:20 ratio, ensuring that images from the same participant were not included in both the training and validation sets. This resulted in 28,063 images for training and 7200 images for validation. The dataset contains five eye-sign directions (center, left, right, up, and down) captured under various lighting conditions, including natural light, artificial light, morning and evening illumination, and left-, right-, and front-facing light sources. In addition, data augmentation techniques such as brightness and contrast adjustment, color jittering, shadow and highlight simulation, and noise injection were applied to improve model robustness. These variations enhance the adaptability of the model to diverse users and real-world operating conditions.

In this system, the deep-learning model YOLOv5 is used for its excellent features, such as high speed and minimal computation for efficient results, in cases of live eye movement video processed on limited-power systems. For further improvement of this module, hyperparameter optimization was performed to enhance the learning rates. This makes the system more efficient at detecting eye movement across varying lighting conditions. There is always confusion during training on these modules, such as image flips, mixup, and mosaic, especially in eye-gaze communication systems, because the eye images are symmetric, and these augmentations confuse the module. Hence, this image flip, mixup, and mosaic augmentations have been turned off during training of Thrivaad, and instead brightness and contrast changes, and color shifts due to side and front light illuminations have been added, so this preserves eye-gaze symmetry and adapts to varying conditions.

**Figure 3:**
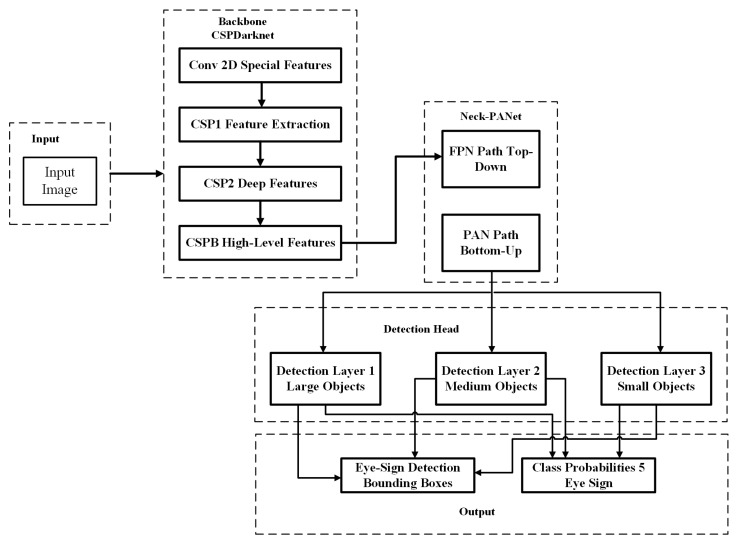
Network architecture diagram of YoloV5.

Figure 3 depicts YOLOv5’s system architecture in this device, which consists of four major blocks, the input layer, backbone, neck, and detection head, and then the output is produced. Let us deep-dive into each block one by one. The input layer captures the eye movements and manages the RGB video frames, and then these frames come to the backbone CSPDarknet (Cross-Stage Partial Networks) block, where the extraction happens in order from low-level details like edges and corners to high-level patterns like eye movement direction and shape of the eye. The middle block is Neck PANet (Path Aggregation Network), which connects the backbone and head blocks. Here, the features are split and combined into two different paths, one top–down (context to details) and another bottom–up (details to context). The head block divides the information into three sections: large, medium, and small objects, and then gives two outputs: the bounding boxes and confidence score for the eye movements for the five directions: left, right, up, down, and center. This entire system performs exactly the same, irrespective of lighting and position changes.

**A.**  **Hyperparameter optimization**

Hyperparameter optimization with Optuna delivers a well-organized and self-regulating strategy for identifying well-suited hyperparameters in machine-learning models. Optuna executes a refined optimization framework that uses techniques like Tree-structured Parzen Estimators (TPE) and Bayesian optimization. These techniques are used to study the hyperparameter search space. The parameters of a search space, like learning rate, batch size, and network architecture configurations, are to be specified, and then Optuna completely estimates various combinations and offers the best-performing one. The optimization process occurs in both an iterative and an adaptive manner. To concentrate on favorable areas of the search space, the algorithm harnesses information from previous trials. This makes it more time-efficient than regular search methods. Hence, the models can accomplish faster convergence and potentially higher predictive accuracy by tuning hyperparameters more effectively. Mathematical expressions of hyperparameter optimization using Optuna are given below.

(1) $$\max A\;=\;f\left(lr0,hsvv,scale,hsvs,anchort,WeightFile\right)$$

The primary function for hyperparameter optimization is given in Equation (1). In this equation: *maxA* is the maximum accuracy, *f* is a function, *lr*0 is the learning rate, *hsvv* and *hsvs* are augmentation parameters, *anchor t* is the anchor threshold, and *weight file* is the pre-trained weight file. So the equation conveys that maximum accuracy is dependent on the function of the following parameters.

**1. Hyperparameter Sampling Space**

Each hyperparameter is set within a boundary range, such as for continuous parameters.

(2) $$\mathrm{Learning}\;\mathrm{Rate}\;\left(lr_{0}\right):\;lr0\in \left[0.001,0.01\right],\;\;\;\;log-scalesampling$$

Equation (2) shows that the learning rate is sampled in the range of small values (0.001) to large values (0.01) to maintain effectiveness in learning without overshooting and to have fine control on small values for better optimization.

(3) $$\mathrm{HSV}\text{-}\mathrm{Value}\;\mathrm{Augmentation}:\;\left(hsv_{-v}\right)\in \left[0.2,0.7\right]$$

Equation (3) shows that the augmentation parameter *hsvv* is sampled within the range 0.2 to 0.7 to maintain brightness augmentation, in a real-time changing-lighting environment.

(4) $$\mathrm{Image}\;\mathrm{Scale}\;(\mathrm{scale}):\;scale\in \left[0.3,0.9\right]$$

Equation (4) shows that the hypermeter for image scaling is in the range of 0.3 to 0.9. because in this range the image will get adjusted to the input images and makes the generalizability easier for varying object sizes.

(5) $$\mathrm{HSV}\text{-}\mathrm{Saturation}\;\mathrm{Augmentation}\;(hsv_{s}):\;hsv_{s}\in \left[0.5,0.9\right]$$

Equation (3) shows that the augmentation parameter *hsvs* is sampled within the range 0.5 to 0.9 to maintain color saturation augmentation, in a real-time color switching environment.

(6) $$\mathrm{Anchor}\;\mathrm{Threshold}\;(anchor_{t}):\;anchor_{t}\in \left[3.0,5.0\right]$$

Equation (6) shows that the hyperparameter threshold anchor is kept in the range of 3.0 to 5.0 to optimize the object detection accuracy for varied structures. Since the anchor threshold impacts the boundary box matching process during training.

(7) Categorical Parameter: Weight File (Weight File): WeightFile∈yolov5m,yolov5s

Equation (7) shows categorical parameters such as the YOLOv5 models YOLO5m and YOLOv5s, which determine the variant of the pre-trained model since YOLOv5m is accuracy-focused and YOLOv5s is known for computational efficiency.

(8) (A)=f(lr0,hsvv,scale,hsvs,anchort,WeightFile)  

Accuracy is denoted as *A*. It is already mentioned that it is dependent on the function of hyperparameters shown in Equation (8). F is a model evaluation function used to train and validate the model using these specified hyperparameters, and the output of this is computed and evaluated as the accuracy.

**2. Optimization Workflow**

To maximize the model’s accuracy (*A*), the Optuna framework dynamically selects hyperparameters. Below is the equation for the hyperparameter of a given trial (*t*).

(9) $$lr_{0}^{\left(t\right)}=trial.suggest-float(lr0,0.001,0.01,log=True)$$

Equation (9) defines the logarithmically arranged range for the learning rate to maintain training stability and ensure fine-grained exploration of smaller values.

(10) $$hsv_{v}^{\left(t\right)}=trial.suggest-float\;\left(hsvv,\;0.2,\;0.7\right)$$

Equation (10) shows the sampled value for the augmentation parameter (*hsv*) to adjust image brightness for a given trial (*t*) to adapt to varying lighting conditions.

(11) $$scale^{\left(t\right)}=trial.suggest-float\;\left(scale,\;0.3,\;0.9\right)$$

Equation (11) shows the image scale parameters that are sampled in the scale of 0.3 to 0.9 for a given trial to adapt to object size variations for improving the generalizability of the model.

(12) $$hsv_{s}^{\left(t\right)}=trial.suggest-float\;\left(hsvs,\;0.5.\;0.9\right)$$

Equation (12) is the sampling augmentation parameter *hsvs* for saturation. The range is set to simulate different color intensities, making the model robust to environmental variations.

(13) $$anchor_{t}^{\left(t\right)}=trial.suggest-float\;\left(anchort,\;3.0,\;5.0\right)$$

Equation (13) represents an anchor threshold sampled to improve the precision of object detection by fine-tuning the bounding box matching criteria.

(14) $$Weight\;File^{\left(t\right)}=trial\;suggest\;categorical\;\left[weight\;file\;\left(yolov5m,\;yolov5s\right)\right]$$

Equation (14) balances accuracy and computational efficiency; the pre-trained weight file is selected categorically across the YOLO models.

The accuracy obtained for the *t*th Optuna trial is computed as a function of the selected hyperparameters:

(15) $$A^{\left(t\right)}\;=\;f\left(lr_{0}^{\left(t\right)},\;hsv_{v}^{\left(t\right)},\;scale^{\left(t\right)},\;hsv_{s}^{\left(t\right)},\;anchor_{t}^{\left(t\right)},\;Weight\;File^{\left(t\right)}\right)$$

Equation (15) defines the validation accuracy *A*^(t)^ obtained during the *t*th Optuna trial as a function of the selected hyperparameters. Here, f represents the YOLOv5 training and evaluation process performed using the specified hyperparameter configuration.

After completing *NN*N optimization trials, Optuna identifies the hyperparameter set that yields the highest validation accuracy:

(16) $$\theta^{*}\;=\;f\left(lr_{0}^{*},\;hsv_{v}^{*},\;scale^{*},\;hsv_{s}^{*},\;anchor_{t}^{*},\;Weight\;File^{*}\right)$$

After completion of N optimization trials, Optuna identifies the best-performing hyperparameter set. Equation (16) represents the optimal hyperparameter configuration θ^*^ that yields the highest validation accuracy among all evaluated trials.

The maximum validation accuracy corresponding to the optimal hyperparameter set is expressed as:

(17) $$A^{*}\;=\;f\left(lr_{0}^{*},\;hsv_{v}^{*},\;scale^{*},\;hsv_{s}^{*},\;anchor_{t}^{*},\;Weight\;File^{*}\right)$$

The maximum validation accuracy obtained using the optimal hyperparameters is denoted by A^*^. Equation (17) computes the highest achievable validation accuracy corresponding to the optimal hyperparameter set θ*. The identified hyperparameters are subsequently used for fine-tuning the final YOLOv5 model.

**B.**  **Thrivaad Patterns**

**Figure 4:**
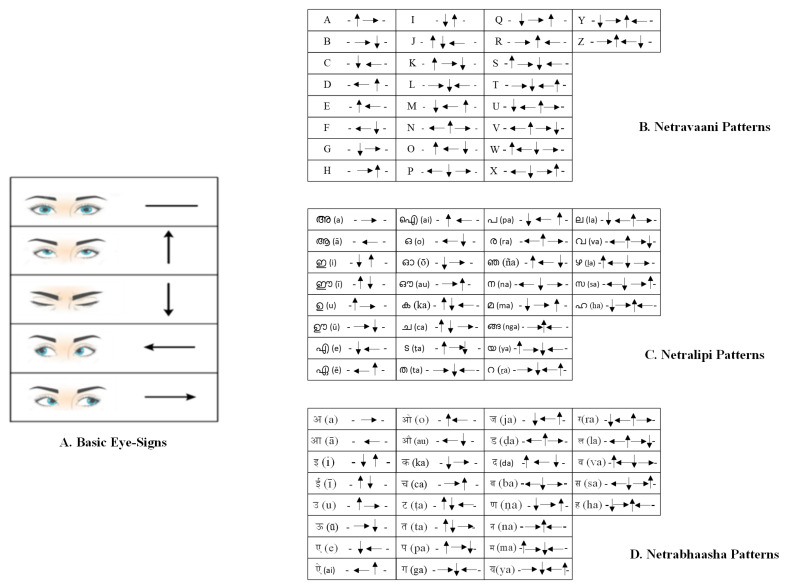
Basic eye signs and Thrivaad patterns.

Thrivaad is an eye-gaze-based communication system that helps people with motor impairments who are unable to communicate verbally. To make a system more user-friendly, Thrivaad introduced non-verbal communication through eye gaze in three languages: Netravani for English, Netralipi for Malayalam, and Netrabhasha for Hindi. Some unique patterns have been made for these languages to create words and sentences for communication. To establish communication, this system uses five eye signs: straight, left, right, up, and down, as shown in Figure 4A. These are very simple and effortless for individuals. Figure 4 shows different eye signs. These eye-sign combinations are Thrivaad patterns and are used to form numbers, words, and sentences in user-defined languages. To remember the words for repeatability, the straight [—] sign is used before and after each word displayed for interaction. Let us consider representing the English alphabet A, the pattern is **“- ↑ → -“** meaning straight, up, right, then straight. So, these kinds of patterns will be followed for the other respective languages’ alphabets, numbers and words.

**C.**  **GUI in Thrivaad**

**Figure 5:**
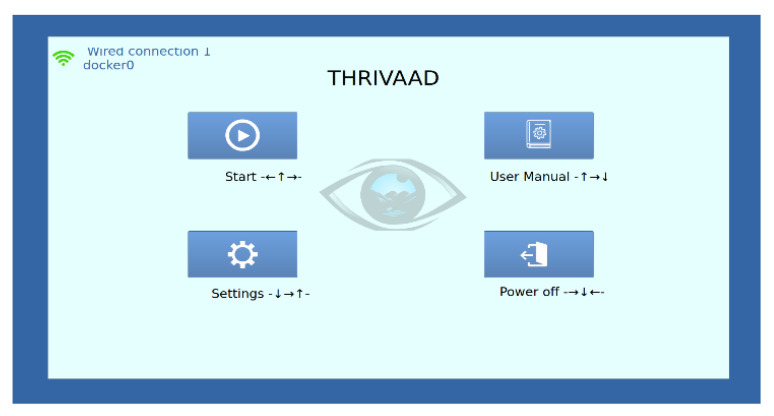
Home page of Thrivaad.

**Figure 6:**
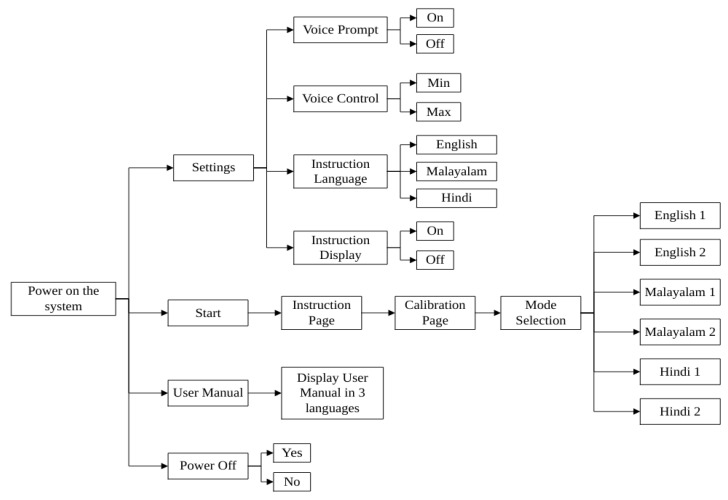
System architecture of Thrivaad GUI.

**Figure 7:**
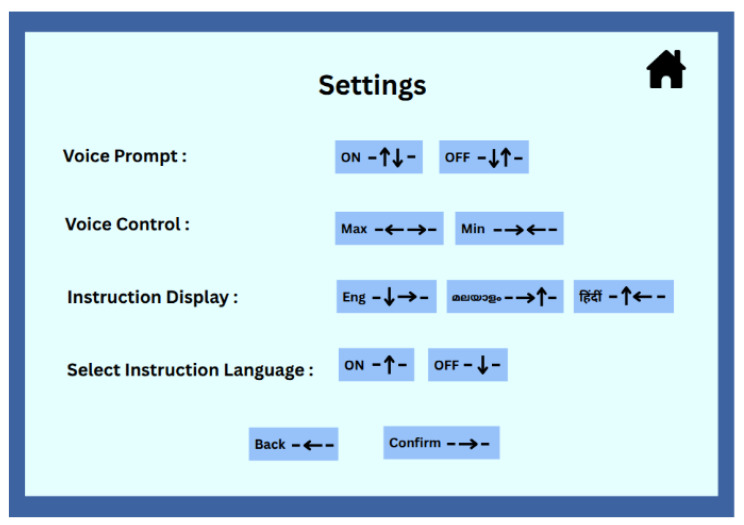
Settings page of Thrivaad.

**Figure 8:**
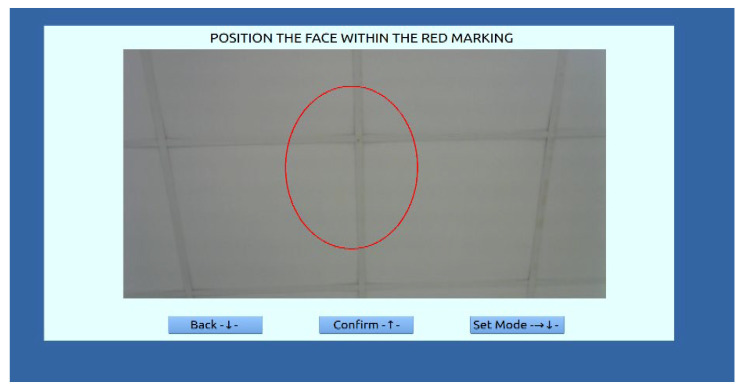
Calibration page of Thrivaad.

The GUI shown in Figure 5, Thrivaad system’s homepage, contains four buttons (start, settings, user manual, power off) with a widget and an eye-sign pattern in it. As illustrated in Figure 6, the eye-sign patterns are specified for each button, so the user can control it as needed, and the system functions accordingly. Figure 7 shows the settings page, which includes the following controls: Voice prompt to enable or disable it, voice control to adjust the voice volume from minimum to maximum, and instruction display to choose the preferred language to be displayed, such as Hindi, Malayalam, and English. To select the instruction language the on and off controls proceed with the selected voice command language. These settings are used to customize the system based on user interests, making it more user-friendly and more adaptable. Once system customization is complete, the user starts the process by selecting the start button on the system’s homepage, which directs the user to the next page where a set of instructions is provided to help the user better utilize the features, such as calibration, mode selection, language, and so on. After the instruction part, the user will be directed to the calibration page, where they can adjust the position of their face to properly align their eyes for accurate detection of the user’s eye signs, as shown in Figure 8. Then, the user will select their preferred mode (six options) and language (three options). This user journey in the GUI makes users more attentive and interactive, helping kick-start communication.

**D.**  **Mode Selection**

**Figure 9:**
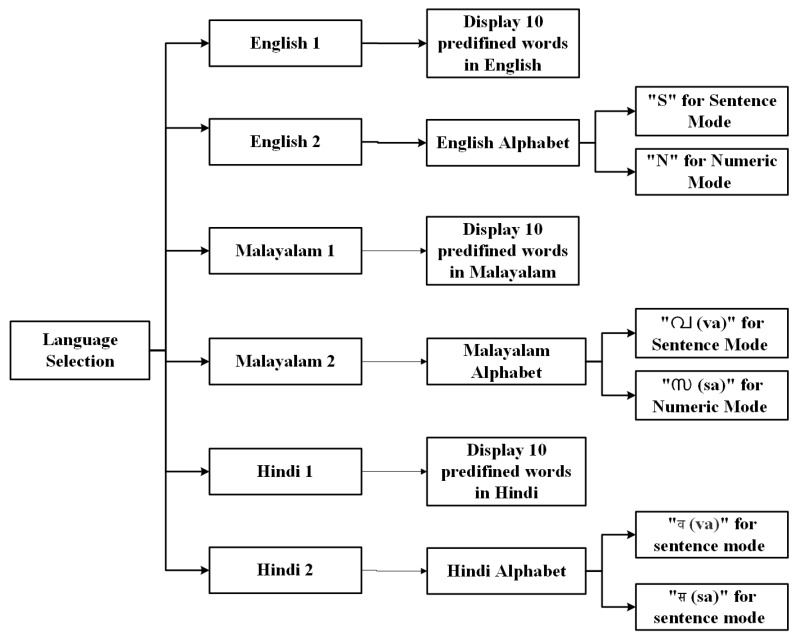
Language selection in Thrivaad.

**Figure 10:**
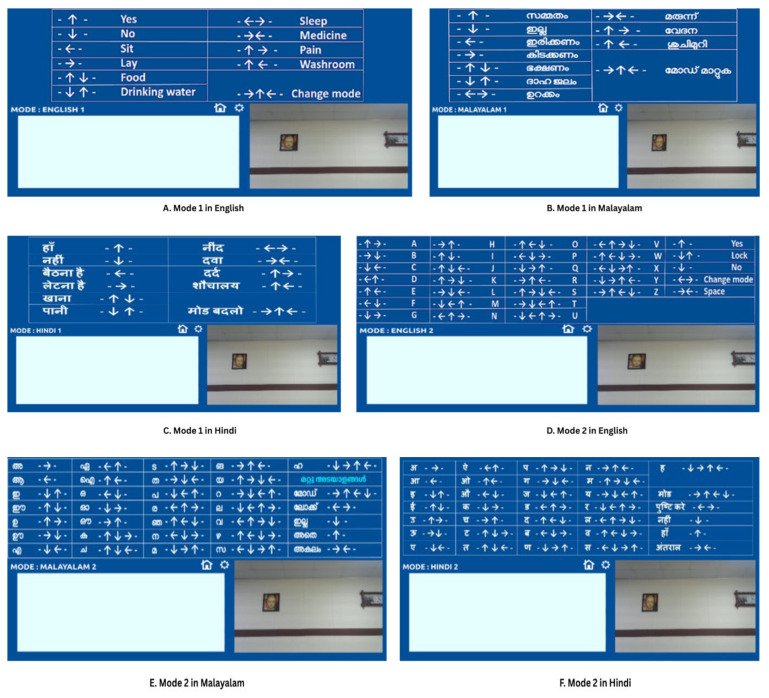
Language Mode 1 and Mode 2.

Continuing the Thrivaad GUI architecture, the language selection architecture shown in Figure 9 illustrates the systematic selection of language modes, since each language has two modes, for example, Malayalam 1 and Malayalam 2, and likewise for English and Hindi. Mode 1 has 10 predefined words of the respective languages, and Mode 2 has the alphabet of the respective languages. For example, a list of 10 predefined words is provided for each language option, as shown in Table 1. To provide a detailed understanding of Mode 1, Figure 10A–C shows the mechanisms for all three languages (Hindi, English, Malayalam). For Mode 2, Figure 10D–F is shown below. This gives the user flexibility, as they can switch between modes and build communication. Apart from the letters, this system has a numeric mode in each language, and switching between them is done using the following controls. For example, for an English user who wants to switch from sentence mode (letters) to numeric (number mode), they have to navigate to mode change, and then there are two options, ‘S‘ and ‘N’. For sentence mode, select ‘S’ and, for numeric mode, select ‘N’; this takes the user to the respective mode. Similarly, the same procedure applies to the other two languages, Malayalam (“വ” and “സ”) and Hindi (“व” and “स”), as shown in Figure 10.

Why are there multiple modes in this multilingual system? Users are of three types: Basic, Intermediate, and Advanced, based on literacy and technological familiarity. So basic users can use predefined words/phrases to convey what they want to talk about, in the case of intermediate users, they can build a sentence using words, and advanced level users can build a unique sentence by creating new words letter by letter and can use numbers as well. So, this system is designed for inclusivity, and hence it is a universally designed GUI, which provides a seamless user experience with no language barrier. This system is primarily designed for non-verbal communication between motor-impaired individuals, and it can be used as a virtual assistant or as an interactive multilingual platform.

While interacting with this system, they can select the word/sentence prediction option to reduce their effort, as this feature predicts the word/sentence the user has in mind based on the initial letters and the sentence’s wording.

For example:

- When the user wants to say “Apple” and starts with the letter “A,” the system auto-suggests words that start with the first letter, such as Apple, Ant, and Arm. The user can select a word from those suggestions.
- If a user wants to say “Mother” in Malayalam and starts with the letter “അ,” this system auto-suggests the following: “അമ്മ” (mother) and “അലങ്കാരം” (decoration). The user can select a word from those suggestions.
- If a user wants to say the word “Good” and starts with the letter “अ,” this system auto-suggests the following: “अग्नि” (fire), “अच्छा” (good) and “अजनबी” (stranger). The user can select a word from those suggestions.

The strategy is to reduce the user’s cognitive load, making typing intuitive and improving the user experience. This makes the system more efficient by saving time and enhancing communication speed.

## 4. Experiments and Results

This dataset is split into an 80:20 ratio for training and validation, that is, 28,063 for training and 7200 for validation out of 35,263 eye-sign images. The YOLOv5 models are trained to minimize loss functions such as box loss (optimizing localization), confidence loss (presence accuracy), and class loss (eye movement direction prediction) using a standard composite loss function. To choose the best YOLOv5 model, the following models are trained and evaluated: YOLOv5n, YOLOv5s and YOLOv5m. To evaluate the final model, the precision metric is used as the gold standard to avoid incorrect eye-gaze direction results; hence, this model is reliable for user interaction. In addition to model evaluation, the proposed Thrivaad system was validated using 60 healthy participants aged between 19 and 65 years. The participants were recruited solely for system validation and usability assessment under controlled experimental conditions. The system was tested in both a simulated clinical environment and a standard office environment established within the workplace setting. Experiments were conducted under different lighting conditions and in both sitting and bed-lying positions to evaluate the robustness, reliability, and usability of the system across diverse AAC usage scenarios.

### 4.1. Eye Movement Detection Using YOLOv5 + Optuna Hyperparameter Optimization

To determine the most optimal architecture among various YOLOv5 model options for eye-sign recognition, three YOLOv5 models, specifically YOLOv5n, YOLOv5s, and YOLOv5m, were trained and evaluated on the constructed eye-sign dataset. This eye-sign dataset, containing a total of 35,263 eye-sign images, was split into an 80:20 training and validation set. During the training process, these models were trained on a loss function composed of box loss, confidence loss, and class loss to optimize eye localization, confidence in detecting eye signs and prediction of eye movement. Evaluating these three models for the task of eye-sign recognition in the Thrivaad system led to results with overall accuracy of 94.26% for YOLOv5m outperforming the others with 90.23% and 88.78% for YOLOv5s and YOLOv5n respectively. The YOLOv5m model provided the optimum balance between feature extraction capabilities and detection accuracy of the eye sign, yielding it to be the most appropriate choice for a robust eye-sign recognition for the Thrivaad system.

In order to further optimize the performance of detection of the eye-sign dataset, a hyperparameter optimization and ablation study was carried out. This study involved modification of specific hyper-parameters including learning rate (lr0), HSV-Value augmentation (hsvv), scale augmentation for the image (scale), HSV-Saturation augmentation (hsvs) and anchor threshold (anchor_t). From Table 2, it can be observed that hyperparameter modification of specific variables can affect the model’s output considerably. YOLOv5m achieved the best validation performance (78.2%) with a learning rate of 0.001, an HSV-Value augmentation of 0.2, a scale augmentation for the image of 0.3, an HSV-Saturation augmentation of 0.5 and anchor threshold of 3.0. In further experiment, it was found that an increase in learning rate and augmentation strength resulted in decreased performance. In fact, YOLOv5m model obtained a low performance of 65.8% with learning rate of 0.01 and greater augmentations. It was also seen that when the same hyperparameter modification was applied to the YOLOv5s model the performance values were even lower, with the maximum value obtained being 60.5%.

**Figure 11:**
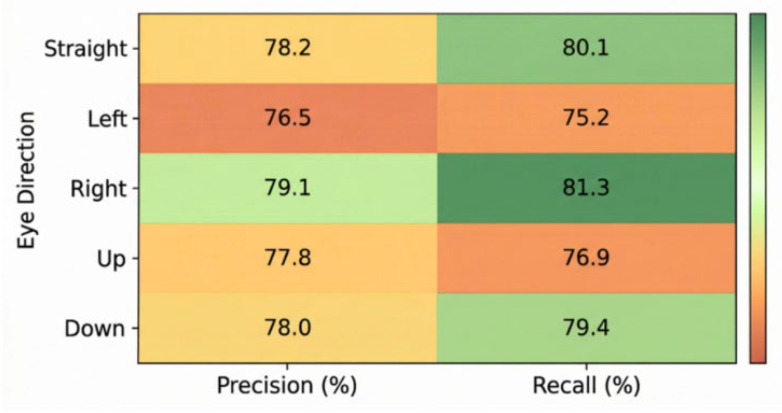
YOLOv5 detection performance. Green shades indicate higher values (better performance). Yellow shades indicate moderate values. Orange to red shades indicate lower values (weaker performance).

After an ablation report, the best configuration was selected and tested, evaluated in each direction of eye movement, and the following results were obtained. Figure 11 shows the results of this experiment, demonstrating that the Thrivaad interface is more reliable for eye-sign detection.

### 4.2. Smart Word Selector for Enhanced Eye-Based AAC

To address the limitation of eye-gaze-based AAC systems’ communication speed, the Thrivaad system has been tested and evaluated for time consumption in effective communication. Table 3 shows a detailed summary of time consumption in all three languages for letter selection. For example, the time taken for English letters is around 4–5 s, while some Malayalam vowels (e.g., “അ (a)” at 3.04 s) and Hindi vowels (e.g., “अ (a)” at 2.78 s). This shows that, regardless of language, the time range is 3–5 s for the individual to create a letter in Thrivaad, as shown in Table 3.

The main cause of slower communication in eye-gaze-based AAC systems is the inability to predict the word or sentence the user wants to convey. If prediction does not occur, the user must type a word letter by letter, which increases cognitive load and eye strain, and eventually, the user will give up on this communication. To address this, testing and evaluation have been conducted and are presented in Table 4. The results are for both modes (predicting and non-predicting), and comparing them confirms that the word-prediction feature enhances communication speed. For example, if a user wants to type “Apple,” it takes 42.69 s to build this word; in prediction mode, it takes 18.26 s. Similarly, time is drastically reduced for the word “Fever” from 44.41 s to 10.17 s.

**Figure 12:**
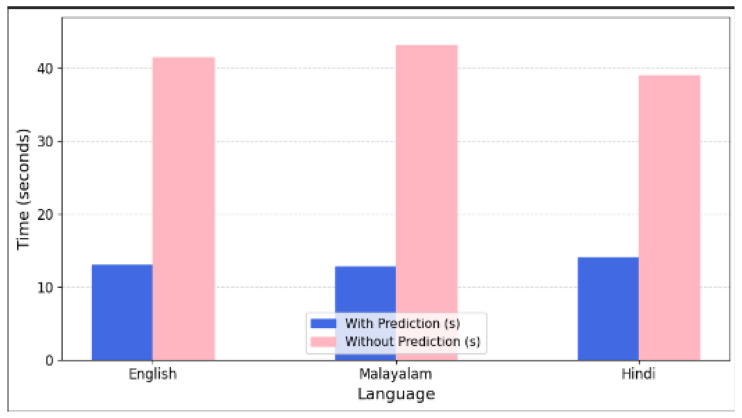
Word creation time.

As summarized in Table 4, the English, Malayalam, and Hindi words are averaged at 13.2 s, 12.8 s, and 14.1 s, respectively, in the word-prediction mode, and without word prediction, the word creation time is averaged for the English, Malayalam, and Hindi words at 41.5 s, 42.9 s, and 39.2 s, respectively. This is shown in Figure 12.

To demonstrate the feasibility of the proposed Thrivaad system to support real-time AAC, evaluation was done based on a confusion matrix for predefined commands in English, Malayalam and Hindi (shown in Table 5, Table 6 and Table 7, respectively). Standard frequently used AAC commands such as ‘Yes’, ‘No’, ‘Sit’, ‘Lay’, ‘Food’, ‘Drinking Water’, ‘Sleep’, ‘Medicine’, ‘Pain’, ‘Washroom’ and ‘Change Mode’ were considered and the TP, TN, FP, FN, precision, recall and accuracy values were calculated to check how reliably the intended commands could be extracted from the eye-sign pattern. The majority of the commands in Table 5, Table 6 and Table 7 give higher precision, recall and accuracy values indicating that most of the commands could be reliably identified from the corresponding eye-sign pattern. The English command set has almost perfect detection accuracy while for Malayalam and Hindi there are some false positive and false negative values for certain commands. Thus, the proposed eye-sign detection and pattern-recognition framework can effectively serve in multilingual AAC.

### 4.3. Multilingual User-Centric GUI for Eye-Based AAC

The results of the user-friendliness survey conducted with 60 participants are summarized in Table 8. Following system validation, participants completed a structured paper-based questionnaire consisting of ten Yes/No questions immediately after interacting with the Thrivaad system. The survey evaluated ease of use, effectiveness of the Smart Word Selector, multilingual GUI accessibility, navigation, voice prompts, user guidance features, eye strain, and overall user satisfaction. Based on the responses, 80% of participants (48 out of 60) found Thrivaad easy to use, while 90% (54 out of 60) reported that the word-prediction feature reduced the effort required to create words and sentences. All participants positively evaluated the multilingual GUI, voice prompts, and overall user experience, indicating improved accessibility and communication effectiveness. Additionally, 90% of participants found the instruction chart and page navigation features helpful during system interaction. Regarding user comfort, 40% of participants reported experiencing mild and temporary eye strain, while 60% reported no noticeable discomfort. Feedback analysis revealed that eye strain was primarily experienced by participants who relied on manual letter-by-letter text generation rather than utilizing the predefined words and Smart Word Selector feature, resulting in longer interaction times and increased visual effort. Participants indicated that the discomfort generally subsided after a short rest period of approximately five minutes. Overall, the survey findings demonstrate that the proposed multilingual GUI, predictive text functionality, and accessibility-oriented design features provide a positive user experience and effectively support the communication needs of individuals who depend on AAC technologies.

## 5. Conclusions and Future Work

Thrivaad is a multilingual eye-sign-based AAC system that uses an inclusive graphical user interface that supports Hindi, Malayalam, and English to facilitate communication for people with speech and mobility impairments. YOLOv5m had the highest eye-sign classification accuracy of 94.26% among the assessed YOLOv5 variations. The model’s performance was further enhanced by hyperparameter optimization, which produced the best validation accuracy of 78.2% with the ideal set of learning rate, augmentation parameters, and anchor threshold. With average word formation times of 13.2, 12.8, and 14.1 s for English, Malayalam, and Hindi, respectively, the Smart Word Selector feature decreased the effort needed for message construction and increased communication speed. A total of 80% of the 60 healthy participants in the usability study said the system was simple to use, and 90% said the word-prediction tool made communication easier. Additionally, participants responded favorably to the audio prompts, navigation tools, and bilingual GUI, indicating the viability and usefulness of the suggested method.

The study also identified several limitations. During usability testing, 40% of participants reported mild and temporary eye strain, particularly during prolonged letter-by-letter text generation without extensive use of the predictive text function. Although the discomfort generally subsided after a short rest period, reducing visual fatigue remains an important area for future improvement. Furthermore, the system currently supports only three languages and can be extended to additional regional languages such as Kannada, Tamil, and Telugu. Another important limitation is that the system validation and usability assessment were conducted using able-bodied participants rather than individuals with speech and motor impairments, who represent the intended end-user population. Therefore, while the results demonstrate the technical feasibility and usability of the proposed system, further evaluation involving users with conditions such as ALS, cerebral palsy, and other severe motor disabilities is required before broader clinical applicability can be established.

Future work will focus on reducing eye strain through improved interface design, expanding multilingual support, enhancing the predictive text engine through adaptive user-specific learning, and improving system deployment using optimized webcam integration and advanced data augmentation strategies. In addition, comprehensive validation with the target clinical population will be conducted to assess long-term usability, communication effectiveness, and real-world applicability of the proposed AAC system.

## Figures and Tables

**Table 1:** Predefined words in each language.

English 1	Malayalam 1	Hindi 1
Yes	സമ്മതം	हाँ
No	ഇല്ല	नहीं
Sit	ഇരിക്കണം	बैठना है
Lay	കിടക്കണം	लेटना है
Food	ഭക്ഷണം	खाना
Drinking Water	ദാഹജലം	पानी
Sleep	ഉറക്കം	नींद
Medicine	മരുന്ന്	दवा
Pain	വേദന	दर्द
Washroom	ശുചിമുറി	शौचालय

**Table 2:** Hyperparameter optimization result.

lr0 (Learning Rate)	hsv_v (HSV-Value Augmentation)	Scale (Image Scale)	hsv_s (HSV-Saturation Augmentation)	Anchor_t	Weight File	Validation Score
0.001	0.2	0.3	0.5	3	yolov5m	78.2
0.005	0.5	0.6	0.7	4	yolov5m	73.4
0.01	0.7	0.9	0.9	5	yolov5m	65.8
0.003	0.4	0.5	0.6	3.5	yolov5m	71.3
0.007	0.6	0.7	0.8	4.5	yolov5m	67.9
0.001	0.3	0.4	0.6	3.2	yolov5s	60.5
0.009	0.7	0.8	0.9	4.8	yolov5s	56.3

**Table 3:** Time taken to create letters in three languages.

Alphabets	Time Taken	Alphabets	Time Taken	Alphabets	Time Taken
A	4.85 s	അ (a)	3.04 s	अ (a)	2.78 s
B	5.27 s	ആ (ā)	3.52 s	आ (ā)	3.23 s
C	5.08 s	ഇ (i)	5.29 s	इ (i)	4.36 s
D	5.28 s	ഈ (ī)	5.13 s	ई (ī)	5.28 s
E	4.61 s	ഉ (u)	4.36 s	उ (u)	4.88 s
F	4.76 s	ഊ (ū)	3.67 s	ऊ (ū)	5.13 s
G	4.88 s	എ (e)	4.52 s	ए (e)	4.61 s
H	3.35 s	ഏ (ē)	4.88 s	ऐ (ai)	4.89 s
I	5.29 s	ഐ (ai)	4.25 s	ओ (o)	5.08 s
J	4.89 s	ഒ (o)	5.21 s	औ (au)	5.13 s

**Table 4:** Time taken to create words with and without word prediction.

Words	Time Taken with Word Prediction	Time Taken Without Word Prediction
Apple	18.26 s	42.69 s
Backache	10.35 s	1 min 01 s
Cough	12.43 s	40.37 s
Doctor	16.34 s	42.46 s
Energy	13.41 s	45.40 s
Fever	10.17 s	44.41 s
Glass	15.51 s	41.21 s
Headache	09.95 s	54.35 s
Ice	10.38 s	17.86 s
Juice	s10.69 s	30.95 s
അകത്ത്	15.25 s	28.51 s
കുറഞ്ഞു	14.26 s	41.66 s
മടക്കുക	11.11 s	53.50 s
എഴുതുക	09.70 s	33.65 s
നന്നായി	13.53 s	47.27 s
വായിക്കുക	14.30 s	57.16 s
താഴെ	13.56 s	30.41 s
ഒന്നുകൂടി	12.21 s	51.94 s
ചെയുക	13.85 s	45.05 s
രാവിലെ	09.38 s	39.35 s
ऊपर	11.64 s	36.33 s
अनार	17.62 s	32.63 s
आराम	12.60 s	33.44 s
पानी	13.31 s	29.82 s
चावल	15.47 s	39.72 s
दबाव	13.81 s	38.42 s
बीमार	16.07 s	38.64 s
तनाव	15.73 s	37.59 s
रहना	14.14 s	40.44 s
नारियल	14.67 s	54.70 s

**Table 5:** Confusion matrix-based performance analysis of English command recognition.

Command	TP	TN	FP	FN	Recall	Precision	Accuracy
Yes	18	0	0	0	100%	100%	100%
No	18	0	0	0	100%	100%	100%
Sit	18	0	0	0	100%	100%	100%
Lay	18	0	0	0	100%	100%	100%
Food	18	0	0	0	100%	100%	100%
Drinking Water	18	0	0	0	100%	100%	100%
Sleep	18	0	0	0	100%	100%	100%
Medicine	18	0	0	0	100%	100%	100%
Pain	17	0	1	0	100%	94%	94%
Washroom	18	0	0	0	100%	100%	100%

**Table 6:** Confusion matrix-based performance analysis of Malayalam command recognition.

Command	TP	TN	FP	FN	Recall	Precision	Accuracy
സമ്മതം	18	0	0	0	100%	100%	100%
അല്ല	18	0	0	0	100%	100%	100%
ഇരിക്കണം	18	0	0	0	100%	100%	100%
കിടക്കണം	18	0	0	0	100%	100%	100%
ഭക്ഷണം	17	1	0	0	100%	100%	100%
ദാഹജലം	18	0	0	0	100%	100%	100%
ഉറക്കം	17	1	0	0	100%	100%	100%
മരുന്ന്	16	1	0	1	94%	100%	94%
വേദന	16	0	0	2	89%	100%	89%
ശുചിമുറി	15	2	0	1	94%	100%	94%

**Table 7:** Confusion matrix-based performance analysis of Hindi command recognition.

Command	TP	TN	FP	FN	Recall	Precision	Accuracy
हाँ	17	0	0	1	94%	100%	94%
नहीं	17	0	0	1	94%	100%	94%
बैठो	18	0	0	0	100%	100%	100%
लेटो	16	0	0	2	89%	100%	89%
खाना	15	0	1	2	88%	94%	83%
पानी	16	0	0	2	89%	100%	89%
सोना	15	1	0	2	88%	100%	89%
दवाई	14	1	0	3	82%	100%	83%
दर्द	13	0	1	4	76%	93%	72%
शौचालय	15	1	0	2	88%	100%	89%

**Table 8:** Result of survey conducted to find user-friendliness.

Questions	Yes	No
Is Thrivaad easy to use?	80%	20%
Did word prediction help to reduce the effort to create a word or sentence?	90%	10%
Is the GUI user friendly?	100%	0%
Do the multiple language options make the system more relevant?	100%	0%
Is the instruction chart feature helpful for a new user?	90%	10%
Is voice prompt helpful for better communication?	100%	0%
Are you able to navigate pages easily using eye signs?	90%	10%
Does the user manual provide clear guidance?	100%	0%
Does making eye signs cause eye strain?	40%	60%
Based on your experience, does it meet the user’s requirements?	100%	0%

## Data Availability

The datasets generated during and/or analyzed during the current study are available from the corresponding author on reasonable request.
